# Does regulation increase the rate at which doctors leave practice? Analysis of routine hospital data in the English NHS following the introduction of medical revalidation

**DOI:** 10.1186/s12916-019-1270-4

**Published:** 2019-02-11

**Authors:** Nils Gutacker, Karen Bloor, Chris Bojke, Julian Archer, Kieran Walshe

**Affiliations:** 1 Centre for Health Economics, University of York, York, UK; 2 Department of Health Sciences, University of York, York, UK; 3 Leeds Institute of Health Sciences, Faculty of Medicine and Health, University of Leeds, Leeds, UK; 4 Collaboration for the Advancement of Medical Education Research Assessment, University of Plymouth, Plymouth, UK; 5 Alliance Manchester Business School, University of Manchester, Manchester, UK

**Keywords:** Medical revalidation, Regulation, Medical workforce, Policy evaluation, Retention

## Abstract

**Background:**

In 2012, the UK introduced medical revalidation, whereby to retain their licence all doctors are required to show periodically that they are up to date and fit to practise medicine. Early reports suggested that some doctors found the process overly onerous and chose to leave practice. This study investigates the effect of medical revalidation on the rate at which consultants (senior hospital doctors) leave NHS practice, and assesses any differences between the performance of consultants who left or remained in practice before and after the introduction of revalidation.

**Methods:**

We used a retrospective cohort of administrative data from the Hospital Episode Statistics database on all consultants who were working in English NHS hospitals between April 2008 and March 2009 (*n* = 19,334), followed to March 2015. Proportional hazard models were used to identify the effect of medical revalidation on the time to exit from the NHS workforce, as implied by ceasing NHS clinical activity. The main exposure variable was consultants’ time-varying revalidation status, which differentiates between periods when consultants were (a) not subject to revalidation—before the policy was introduced, (b) awaiting a revalidation recommendation and (c) had received a positive recommendation to be revalidated. Difference-in-differences analysis was used to compare the performance of those who left practice with those who remained in practice before and after the introduction of revalidation, as proxied by case-mix-adjusted 30-day mortality rates.

**Results:**

After 2012, consultants who had not yet revalidated were at an increased hazard of ceasing NHS clinical practice (HR 2.33, 95% CI 2.12 to 2.57) compared with pre-policy levels. This higher risk remained after a positive recommendation (HR 1.85, 95% CI 1.65 to 2.06) but was statistically significantly reduced (*p* < 0.001). We found no statistically significant differences in mortality rates between those consultants who ceased practice and those who remained, after adjustment for multiple testing.

**Conclusion:**

Revalidation appears to have led to greater numbers of doctors ceasing clinical practice, over and above other contemporaneous influences. Those ceasing clinical practice do not appear to have provided lower quality care, as approximated by mortality rates, when compared with those remaining in practice.

**Electronic supplementary material:**

The online version of this article (10.1186/s12916-019-1270-4) contains supplementary material, which is available to authorized users.

## Background

Systems of medical regulation exist around the world, to protect patients and assure the public of the competence and quality of medical practitioners. Traditionally, medicine, like other learned professions, has regulated itself, setting and monitoring high standards of education, controlling entry into the profession and encouraging ethical behaviour based on underlying altruistic principles. Confidence in the medical profession ranks higher than many other areas of life [1], but the public trust upon which self-regulation relies has apparently been eroded in recent years, in all social institutions, including health care systems [2]. Regulatory systems have emerged as a result of this erosion of trust, but these have also come under scrutiny: the ‘quest for accountability’ has resulted in detailed control and a change in culture and performance management which may be viewed as ‘distorting the proper aims of professional practice and indeed as damaging professional pride and integrity’ [3]. Balancing public protection with professional respect in regulating medicine is difficult but essential.

In the UK, public outcry over some failures of medical regulation (most notably errors by paediatric cardiac surgeons in Bristol [4] and the activities of a prolific serial killer in general practice [5]) resulted in reforms of previous regulatory processes. The governance of the national medical regulator, the General Medical Council (GMC), was reformed to make council members appointed rather than elected and to increase lay representation. Self-regulation was widely deemed ineffective, and the profession left ‘fatally vulnerable to the problem of “bad apples”: those unwilling, incapable or indifferent to delivering on their professional commitments and who betrayed the trust of both patients and peers’ [6].

Introduced in December 2012, medical revalidation is a process by which all practising doctors in the UK now have to show periodically that they are up-to-date and fit to practise medicine. Revalidation was viewed as a ‘historic’ change, which would ‘make a major contribution to the quality of care that patients receive’ and ‘give them valuable assurance that the doctors who treat them are regularly assessed’ (Sir Peter Rubin, quoted in [7]). Its development has been closely observed by international medical regulators [8], and those regulating other health professions such as nursing and midwifery [9]. Its policy objectives are ‘trust, assurance and safety’ [10]: maintaining and improving public confidence in the profession, assuring the general public of the quality of medical care they are likely to receive, improving the quality of medical performance, assessing the fitness to practise of individual doctors and ensuring early detection of individuals who are failing to provide safe and effective clinical care. Revalidation applies to all doctors who wish to retain a licence to practise in the UK. The process does not include standardised tests, but involves a system of annual appraisal, maintenance of a portfolio of supporting information and a review and revalidation recommendation, made by a ‘responsible officer’ (RO) usually every 5 years. Supporting information includes data on activities such as continuing professional development, quality improvement, significant events and learning from them, and feedback from patients and colleagues, including complaints and compliments [11]. Time and, to a lesser degree, financial costs may be considerable. The RO is usually a senior doctor, who makes revalidation recommendations to the GMC. Doctors are attached to a ‘designated body’ and to a RO—in hospital medicine this would often be a medical director, but in general practice and other settings, arrangements are made through clinical commissioning groups or directly with the GMC. This system means that doctors report to other doctors, which maintains professional control of the regulatory process. Appraisals have been a contractual obligation in the National Health Service (NHS) since 2003, but before 2012 they were less standardised, and implementation had been slow: annual appraisal rates for hospital consultants in England were 64% in 2010 and 88% in 2015/16 [12].

Revalidation recommendations can result in three possible outcomes: (1) the doctor is revalidated, (2) the doctor’s revalidation is deferred until a later date or (3) the doctor does not engage with the revalidation process and is given a notice of non-engagement (GMC 2012). Out of the three outcomes, only deferral and non-engagement pose a theoretical threat to a doctor’s licence; however, as a wide range of circumstances may also lead to a deferral, a deferral decision is viewed by the GMC as a neutral act [13].

Since before its introduction, there have been concerns about the administrative and emotional burden of medical revalidation [14]. There were early reports that some doctors, particularly older doctors, found the process overly onerous and chose to leave practice rather than complete it [15]. A qualitative study in general practice reported some doctors describing it as ‘the final straw’ that prompted a decision to retire [16]. A recent survey of all UK doctors revealed scepticism about whether revalidation led to improved patient safety or identifying doctors in difficulty early, and re-iterated concerns about time costs and administrative burden [17]. On average, respondents reported spending 26.4 h preparing for and attending their most recent appraisal. A policy review published in early 2017 explored this further, confirming that revalidation feels ‘burdensome and ineffective to some doctors’ (p.27), and reporting that individual doctors and their representative bodies have expressed a view that some doctors have relinquished their licence purely because they do not want to meet the requirements of revalidation [12].

All doctors practising in UK medicine must have a licence to practise with the General Medical Council (GMC), but the reverse is not the case: not all doctors on the register are currently in clinical practice. Many doctors who no longer practise may have chosen to keep their licence in the past, for various reasons. At present, around 280,000 doctors are on the register [18] and 140,000 doctors are employed by the UK NHS [19]. Figures released by the GMC noted that in the 3 years before the introduction of medical revalidation (November 2009 to December 2012), 7994 doctors relinquished their licence to practise, and in three and a half years following its introduction (December 2012 to July 2016), this figure was 33,148 (+ 256%) [20]. It is important to note that this may not be actively practising doctors leaving the profession, but if they are no longer practising, they are likely to have been prompted to relinquish this by the introduction of revalidation. From the GMC register, there is no way of separating practising clinicians (in the NHS or elsewhere) from those who no longer practise in any clinical setting but, nevertheless, in the past, still retained a licence.

Our study used activity and mortality data from hospitals in the English NHS to assess the effect of revalidation on the number of doctors who ceased NHS clinical activity, focusing on whether revalidation prompted consultants to cease NHS practice, and whether this is restricted to older age groups. In subsequent analysis, we tested whether the likelihood of ceasing practice as a result of medical revalidation was associated with consultant performance as measured by case-mix-adjusted 30-day mortality rates.

## Methods

### Study design and data sources

We undertook a retrospective cohort study analysing clinical activity and outcome data from English Hospital Episode Statistics (HES) for the period April 2009 to March 2016. The cohort comprised all consultants who were responsible for episodes of NHS-funded inpatient care in English hospitals in the financial year 2008/09 (1 April to 31 March). The unit of recording in the HES dataset is the finished consultant episode (FCE, a period that a patient spends in the continuous care of a consultant), which is assigned to consultants based on their person-identifiable consultant code recorded in HES, which links to their GMC number. Primary speciality was derived from HES and was coded as medical, surgical or other.

Data on consultants’ age and gender, the date of their full registration with the GMC and information on the dates of revalidation meetings and ROs’ subsequent recommendations were requested from the GMC. These data were provided for all consultants that held a period of registration between April 2008 and July 2016. Additional information on consultants’ current employment status was derived from the electronic staff record (ESR) system, and these data were provided by the NHS Digital Organisation Data Service (ODS). The ESR covers NHS Trusts but not independent providers. We used the latest release of the ESR data from February 2017.

Information on 30-day mortality after admission for all patients who received treatment during the study period was provided by the Office for National Statistics.

We excluded consultants from analysis if their consultant code recorded in HES could not be matched against the GMC specialist register or the GMC register was otherwise incomplete, or if they were responsible for fewer than 52 patient episodes (FCEs) (i.e. one per week on average) during the period April 2008 to March 2009.

### Risk of ceasing NHS clinical practice

#### Outcome definition

The primary outcome was end of clinical activity in the English NHS for any reason (referred to from here as ‘exit’). Consultants were deemed to be clinically active at any given date if they took responsibility for at least one FCE on this or any subsequent date until the end of the data period (31 March 2016). Consultants were also deemed to be clinically active until after the end of the data period if we found them to be employed by an NHS Trust in February 2017 (from the most recent available electronic staff record), which accounts for prolonged absences due to, for example, maternity leave or research leave.

#### Hypotheses

The policy intervention was the introduction of mandatory medical revalidation in December 2012. Doctors were informed about the scheduled date of their revalidation at approximately the same time.

**Fig. 1:**
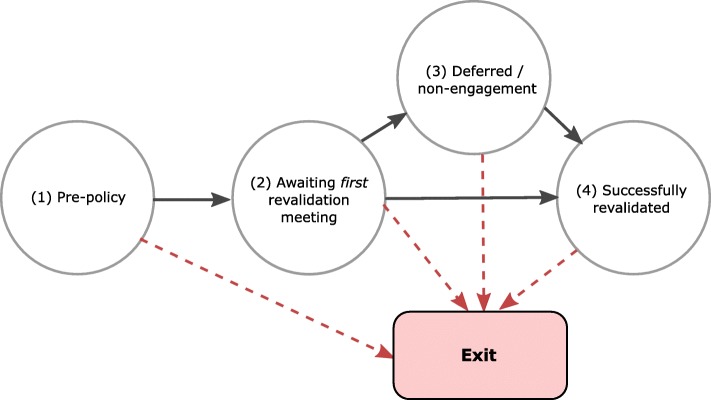
Revalidation states and transition pathways

We hypothesised that the effect of the policy would be to increase the hazard of exit and that this effect would differ according to individual consultants’ revalidation status, which could vary over time and take four different forms (Fig. 1 illustrates the possible transition pathways graphically):

1. Pre-policy implementation—consultant is not subject to revalidation (before December 2012)
2. Post-policy—consultant is preparing for the first revalidation
3. Post-policy—a recommendation has been made to defer the revalidation decision, or the RO has reported non-engagement
4. Post-policy—a recommendation has been made that the consultant should be revalidated

We further hypothesised that the hazard of exit would return to pre-policy levels once consultants received a recommendation that they should be revalidated.

#### Statistical analysis

Semi-parametric Cox proportional hazard models were estimated to identify the effect of medical revalidation on the time to exit. The policy intervention was modelled as a set of time-varying covariates that segregated the post-policy period according to consultants’ revalidation status (preparation, deferral or failure to engage, positive recommendation). The coefficients associated with these covariates measure the change in hazard from the pre-policy period.

We measured time at risk as days from the date the consultant first held a full registration with the GMC. This variable was left-truncated (delayed entry) since we considered only consultants that had not exited before 1 April 2009. Follow-up was until 31 December 2015 to ensure that brief absences around the end of the data period (March 2016), for example due to annual leave, would not be labelled erroneously as exit (this is relevant only for consultants that were not employed by an NHS Trust in February 2017). Data were analysed until exit or the end of follow-up (right-censoring).

Our primary analysis adjusted for factors that may be associated with a risk of leaving practice: consultants’ age at start of follow-up (5-year bands with separate categories for ≤ 40 and > 65), sex, age-sex interactions, whether they received their primary medical qualification outside the UK, main speciality of activity in 2008/09 (coded as medical, surgical and other), volume of activity (number of FCEs) in 2008/09 (coded as 52–200, 201–500, 501–1000, > 1000) and an interaction between speciality and volume. We also performed stratified analyses by age group, gender and training location.

We assessed the robustness of our results through several sensitivity analyses. First, we restricted follow-up to the first year after the policy introduction, i.e. until 30 November 2013. Second, we based outcomes on HES data only (not including electronic staff record data). In this case, we followed consultants until 31 March 2015 to allow for prolonged (1-year) periods of absence. Finally, we estimated parametric survival models covering a range of distributional assumptions.

Estimates are reported as hazard ratios (HRs) with associated 95% confidence intervals (CIs). Values larger than 1 indicate increased risk of exit. Standard errors were clustered at a hospital level. All analyses were performed in Stata version 14 (StataCorp, College Station, TX, USA).

### Change in the clinical performance of consultants ceasing activity

#### Outcome definition

The outcome of interest was patient mortality within 30 days of admission. The analysis was restricted to the last FCE within a patient’s hospital stay, and the outcome was therefore assigned to the last consultant providing care as part of the hospital stay. In sensitivity analysis, we assigned outcomes to the first consultant providing care during the hospital stay.

#### Hypothesis

Medical revalidation may influence poorer performing consultants differently compared with higher performing clinicians. If they believe their own performance is lower than the standard required, they may choose to cease practice if they judge the high effort required to achieve improvements in care quality to outweigh the benefits of retaining a licence. Alternatively, poorer performers may hold overly favourable views of their own ability [21]. We therefore hypothesise that differences in mortality rates between groups of consultants who cease NHS practice (‘leavers’) and those who remain (‘stayers’) could change following the introduction of medical revalidation.

#### Statistical analysis

To test whether the policy intervention is associated with a change in the performance of consultants ceasing activity (as measured by their 30-day mortality rates), we adopted a difference-in-differences (DID) design using ‘stayers’ as a control group. This accounts for contemporaneous changes in medical technology and other external pressures on the health system that apply to ‘leavers’ and ‘stayers’ alike. We estimated separate logistic regression models of 30-day mortality in NHS-funded patients aged 60–89 treated in the pre-policy period (April 2010 to March 2012) and the post-policy period (April 2013 to March 2014). In each model, we included an indicator variable for consultants ceasing clinical activity during this time period. We used the resulting coefficient estimates to calculate the average marginal effect of leaver/stayer status over the entire patient population in that period. The DID estimate denotes the difference between these effects and reflects the change in this performance gap over time that is associated with medical revalidation. If the gap increases after the introduction of revalidation, this would suggest that the group of doctors leaving practice include a higher proportion of poorer performers. These analyses were performed separately for consultants working in medical and surgical specialties. Consultants working in other specialties were excluded because their patients’ profiles were deemed too heterogeneous for meaningful comparison of outcomes.

All regressions models also adjust for a range of patient characteristics, including patients’ age (in 5-year bands), sex, number of Elixhauser co-morbid conditions (coded as 0, 1, 2–3, 4–6, ≥ 7), an indicator of any emergency hospital admissions in the past 365 days, and the Healthcare Resource Group (HRG; the English equivalent of DRGs) root to which the patient had been allocated. The latter adjusts for differences in case-mix and inherent mortality risk across consultants working in different specialties.

Estimates are reported as (differences in) mortality rates with associated 95% confidence intervals (CIs). Standard errors were clustered at a hospital level. All analyses were performed in Stata version 14 (StataCorp, College Station, TX, USA).

## Results

### Risk of ceasing NHS clinical practice

**Fig. 2:**
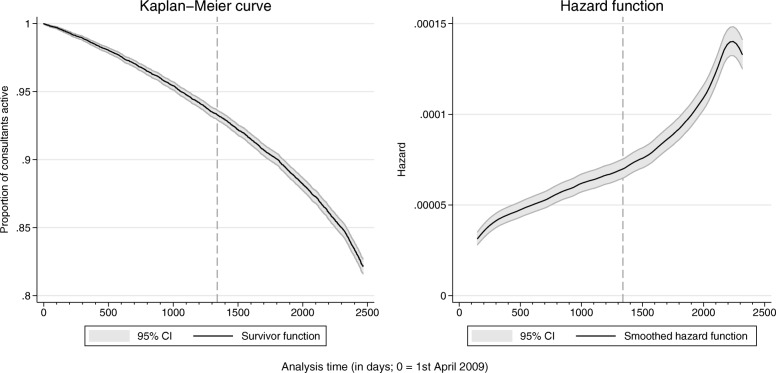
Kaplan-Meier survival function and hazard function, with 95% confidence intervals

**Fig. 3:**
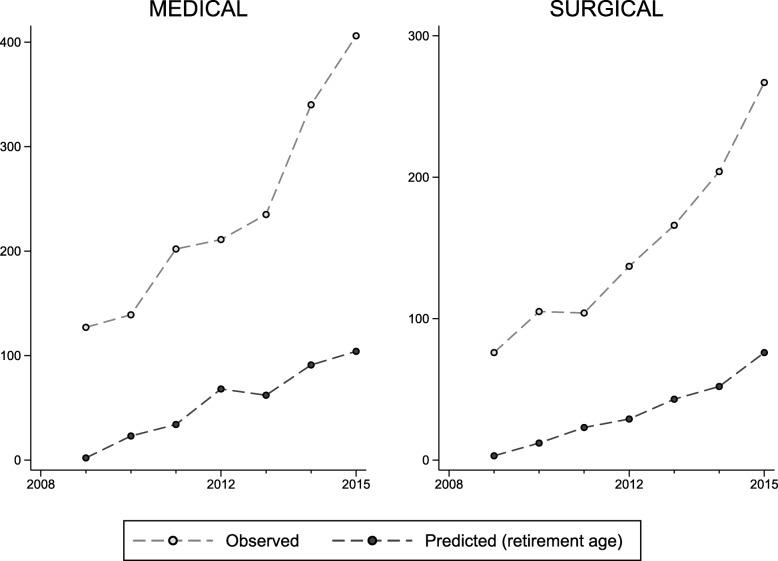
Number of consultants ceasing activity; observed vs. predicted based on retirement at age 65

A total of 29,387 unique consultant codes were recorded in the HES dataset in the period 1 April 2008 to 31 March 2009. Of these, 4392 could not be linked to the GMC register or the register was incomplete, and 5661 were responsible for fewer than 52 FCEs. These consultant codes and the associated clinical activity were excluded from analysis. The remaining 19,334 consultants were followed from 1 April 2009 for a total of 44.4 million days (Table 1). The median follow-up was 2465 days, around 6.7 years (mean = 2298 days, 6.3 years). Approximately 17.9% of consultants (*n* = 3452) ceased to be clinically active before the end of the data analysis period. Of these, 19.9% (*n* = 689) had received a positive revalidation recommendation prior to exit. Figure 2 shows the Kaplan-Meier survival function and the associated hazard function for the cohort of consultants. The vertical dashed line indicates the introduction of medical revalidation in December 2012. Figure 3 shows the cumulative number of consultants ceasing clinical activity over time and the number of those that would have been expected to leave because they reached retirement age (65 years). The difference between the observed and predicted number of exits appears to grow after the introduction of medical revalidation.

**Table 1:** Time spent in each of the revalidation states

Revalidation status	Number of consultant-years in revalidation state	
	Overall	Per consultant
Pre-revalidation	68,866	3.6
Policy in place	29,736	1.5
Deferred/non-engagement	918	0.0
Revalidated	22,215	1.1
Total	121,735	6.3

For the cohort as a whole, the proportion of consultants who received a positive revalidation recommendation increased steadily by approximately 1.9% per month after the policy introduction and reached 85.3% by December 2015. ROs issued a recommendation to defer or reported non-engagement for 1816 consultants, of which 1278 subsequently received a positive recommendation. The median deferral period was 147 days (interquartile range = 113 to 273).

Table 2 presents the final revalidation status at the end of follow-up or time of exit. Consultants who ceased clinical activity before the end of the follow-up period were less likely to have received a positive revalidation recommendation than those that continued clinical activity (38.2% vs. 96.3%, *χ*^2^(1) = 7941; *p* < 0.001). The proportion of consultants ceasing practice after a decision had been deferred is over twice that measured at the end of follow-up (3.9% vs. 1.6%, *χ*^2^(1) = 77.6; *p* < 0.001).

**Table 2:** Last revalidation recommendation at exit or end of follow-up

	Consultants ceasing clinical activity		Consultants continuing clinical activity until end of follow-up		Difference	
Revalidation recommendation	*N*	%	*N*	%	*χ*^2^(1)	*p* value
Awaiting recommendation	1991	57.70%	322	2.00%	8337.9	< 0.001
Revalidate	1319	38.20%	15,301	96.30%	7941.2	< 0.001
Defer	134	3.90%	250	1.60%	77.6	< 0.001
Non-engagement	8	0.20%	9	0.10%	9.9	0.002
Total	3452		15,882			

Table 3 presents the results of the analysis of risk of exit, together with descriptive statistics of the sample. Consultants awaiting their first revalidation recommendation were at higher risk of exit than before they became subject to revalidation (HR 2.33; 95% CI 2.12 to 2.57), and the hazard further increased after a recommendation to defer or a report of non-engagement (HR 3.51; 95% CI 2.71 to 4.55) (*χ*^2^(1) = 10.19; *p* = 0.001). A positive recommendation was also associated with an increased risk of exit compared with pre-policy levels (HR 1.85; 95% CI 1.65 to 2.06) but the hazard was statistically significantly lower than while awaiting the first revalidation meeting (*χ*^2^(1) = 24.36; *p* < 0.001).

**Table 3:** Association between consultants’ characteristics, revalidation status and hazard of exit

Variable	*N* (%)	HR	95% CI
Specialty			
Medical	10,567 (54.7)	(base category)	
Other	1332 (6.9)	1.76	(1.38 to 2.23)
Surgical	7435 (38.5)	1.76	(1.34 to 2.31)
Volume of activity in 2008			
53–99	1424 (7.4)	(base category)	
100–199	1829 (9.5)	0.81	(0.67 to 0.98)
200–299	1542 (8.0)	0.84	(0.67 to 1.06)
300–399	1576 (8.2)	0.71	(0.57 to 0.89)
400–499	1510 (7.8)	0.94	(0.74 to 1.18)
≥ 500	11,453 (59.2)	0.63	(0.53 to 0.74)
Volume × specialty			
Other × 100–199	305 (1.6)	1.30	(0.92 to 1.83)
Other × 200–299	117 (0.6)	0.80	(0.53 to 1.20)
Other × 300–399	68 (0.4)	0.66	(0.34 to 1.27)
Other × 400–499	43 (0.2)	0.74	(0.37 to 1.48)
Other × ≥ 500	303 (1.6)	0.42	(0.28 to 0.61)
Surgical × 100–199	427 (2.2)	0.85	(0.61 to 1.19)
Surgical × 200–299	619 (3.2)	0.71	(0.50 to 0.99)
Surgical × 300–399	759 (3.9)	0.68	(0.48 to 0.96)
Surgical × 400–499	814 (4.2)	0.42	(0.30 to 0.60)
Surgical × ≥ 500	4615 (23.9)	0.54	(0.41 to 0.72)
Country of primary medical qualification			
UK trained	13,487 (69.8)	(base category)	
Foreign trained	5847 (30.2)	1.29	(1.16 to 1.43)
Consultant age (in 2008)			
≤ 40	3336 (17.3)	(base category)	
41–45	5032 (26.0)	1.17	(0.98 to 1.40)
46–50	4250 (22.0)	1.28	(1.05 to 1.56)
51–55	3259 (16.9)	1.76	(1.42 to 2.19)
56–60	2172 (11.2)	2.67	(2.09 to 3.42)
61–65	1117 (5.8)	3.42	(2.58 to 4.54)
> 65	168 (0.9)	3.04	(2.11 to 4.39)
Consultant gender			
Male	15,386 (79.6)	(base category)	
Female	3948 (20.4)	0.90	(0.70 to 1.14)
Revalidation status			
Pre-policy—not subject to revalidation		(base category)	
Post-policy—awaiting revalidation		2.33	(2.12 to 2.57)
Post-policy—deferred/non-engagement		3.51	(2.71 to 4.55)
Post-policy—revalidated		1.85	(1.65 to 2.06)

The hazard of exit was independently associated with consultants’ age (older doctors had a higher risk of exit) and was also higher for non-UK trained doctors compared with those who trained in UK medical schools (HR 1.29; 95% CI 1.16 to 1.43). Consultants working in surgical specialties were at higher risk of exit than those working in medical specialties (HR 1.76; 95% CI 1.34 to 2.32). Risk of exit was negatively associated with volume of activity in 2008 for all specialties (see Additional file 1).

Stratified analyses by age group, gender or country of primary medical qualification confirm the findings of the main analysis and show an increased risk of exit for consultants awaiting their first revalidation meeting (Table 4). The difference in the hazard of exit when awaiting revalidation and after having successfully revalidated is not statistically significant different for women (*p* = 0.082), or consultants aged 51–55 (*p* = 0.464), 61–65 (*p* = 0.136) or > 65 (*p* = 0.810) at the beginning of follow-up. Full regression results are presented in Additional files 2, 3, 4 and 5.

**Table 4:** Stratified analyses of association between consultants’ revalidation status and hazard of exit and sensitivity analyses based on alternative modelling approaches. All regression models adjust for consultant characteristics (not reported)

	*N*	(2) Post-policy—awaiting revalidation		(3) Post-policy—deferred/non-engaged		(4) Post-policy—revalidated		Difference (2)–(4)	
		HR	95% CI	HR	95% CI	HR	95% CI	*χ* ^2^	*p* value
Stratified analysis									
By consultant age									
≤ 40	3336	1.94	(1.42 to 2.64)	0.87	(0.13 to 5.99)	1.02	(0.67 to 1.56)	9.96	0.002
41–45	5032	2.73	(2.12 to 3.52)	6.55	(3.67 to 11.69)	1.30	(0.91 to 1.87)	23.37	< 0.001
46–50	4250	2.85	(2.21 to 3.66)	5.53	(2.65 to 11.52)	2.12	(1.55 to 2.90)	4.96	0.026
51–55	3259	2.52	(1.97 to 3.23)	3.26	(1.70 to 6.27)	2.35	(1.82 to 3.05)	0.54	0.464
56–60	2172	1.94	(1.62 to 2.34)	2.92	(1.79 to 4.75)	1.35	(1.09 to 1.68)	15.73	< 0.001
61–65	1117	1.88	(1.55 to 2.29)	1.47	(0.76 to 2.81)	1.57	(1.20 to 2.04)	2.23	0.136
> 65	168	2.45	(1.68 to 3.56)	3.81	(0.99 to 14.69)	2.24	(1.02 to 4.89)	0.06	0.810
By consultant gender									
Male	15,386	2.41	(2.18 to 2.67)	3.43	(2.58 to 4.58)	1.90	(1.68 to 2.14)	21.98	< 0.001
Female	3948	2.01	(1.63 to 2.48)	3.67	(2.04 to 6.61)	1.60	(1.23 to 2.09)	3.02	0.082
By country of primary medical qualification									
UK trained	13,487	2.07	(1.84 to 2.33)	2.71	(1.95 to 3.76)	1.60	(1.38 to 1.84)	19.84	< 0.001
Foreign trained	5847	2.49	(2.17 to 2.86)	4.66	(3.15 to 6.90)	1.89	(1.57 to 2.28)	11.28	< 0.001
Sensitivity analyses									
Follow-up until 30/11/2014 (based on HES + ESR)	19,334	2.08	(1.90 to 2.27)	2.95	(2.08 to 4.18)	1.73	(1.54 to 1.95)	10.18	0.001
Follow-up until 31/03/2015 (based on HES + ESR)	19,334	1.98	(1.80 to 2.18)	2.53	(1.72 to 3.72)	1.37	(1.20 to 1.57)	32.80	< 0.001
Follow-up until 31/03/2015 (based on HES only)	19,334	1.61	(1.43 to 1.81)	0.57	(0.08 to 4.13)	1.14	(0.83 to 1.57)	4.10	0.043
Parametric model—Exponential	19,334	2.67	(2.44 to 2.91)	4.14	(3.23 to 5.29)	2.20	(1.98 to 2.45)	15.72	< 0.001
Parametric model—Weibull	19,334	2.67	(2.43 to 2.92)	4.14	(3.23 to 5.30)	2.21	(1.97 to 2.47)	15.70	< 0.001
Parametric model—Gompertz	19,334	2.38	(2.17 to 2.62)	3.63	(2.82 to 4.67)	1.89	(1.69 to 2.12)	23.02	< 0.001
Parametric model—Log-Normal	19,334	0.36	(0.26 to 0.49)	0.15	(0.07 to 0.31)	0.50	(0.39 to 0.64)	20.40	< 0.001
Parametric model—Log-Logistic	19,334	0.72	(0.69 to 0.76)	0.58	(0.49 to 0.67)	0.83	(0.79 to 0.87)	39.23	< 0.001

Results were robust to sensitivity analysis using alternative modelling choices including the definition of the outcome and the follow-up period, and to parametric modelling of the hazard function (Table 4).

### Change in the clinical performance of consultants ceasing activity

**Fig. 4:**
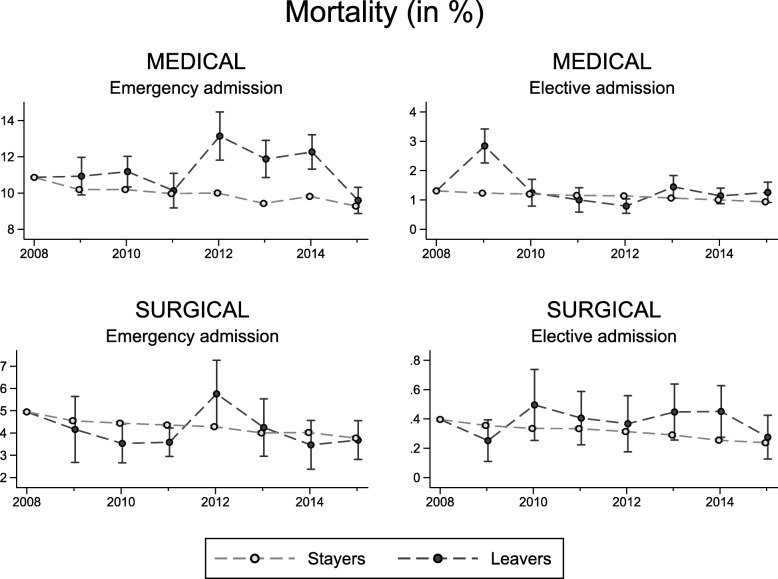
Thirty-day mortality rates (95% CI) of stayers and leavers, 2008 to 2014

Figure 4 shows unadjusted 30-day mortality rates for ‘leavers’ and ‘stayers’, by speciality, admission type and financial year. Table 5 reports group differences for the pre- and post-policy periods (see Additional file 6 for a tabulation of (un) adjusted rates by group and period). There was no statistically significant difference in risk-adjusted mortality rates between ‘leavers’ and ‘stayers’ during the pre-policy period. Risk-adjusted mortality rates improved over time for patients treated by ‘stayers’ but remained largely constant for patients treated by ‘leavers’, thus indicating an increasing performance gap between these groups. However, improvements were generally small and only the DID estimate for the group of elective admissions in surgical specialities was statistically significant (*p* = 0.048). None of the differences remain statistically significant once we apply a Bonferroni correction to counteract the problem of multiple comparisons (which results in a critical value of 0.05/4 = 0.0125). Very similar results were obtained when allocating patients to the first consultant in their hospital stay (see Additional file 7).

**Table 5:** Difference in mortality rates between leavers and stayers by specialty and time period

Index admission	Difference pre-policy			Difference post-policy			Difference-in-difference		
	Est	SE	*p* value	Est	SE	*p* value	Est	SE	*p* value
Unadjusted comparison									
*Consultants working in medical specialties*									
Emergency	0.001	0.002	0.498	0.013	0.002	< 0.001	0.012	0.003	< 0.001
Elective	− 0.0001	0.001	0.856	0.002	0.001	< 0.001	0.002	0.001	0.015
*Consultants working in surgical specialties*									
Emergency	− 0.006	0.002	< 0.001	0.002	0.003	0.430	0.008	0.003	0.011
Elective	0.000	0.000	0.491	0.001	0.000	0.029	0.000	0.001	0.403
Risk-adjusted comparison									
*Consultants working in medical specialties*									
Emergency	− 0.0014	0.004	0.733	0.009	0.008	0.268	0.011	0.009	0.252
Elective	− 0.00026	0.002	0.887	0.003	0.003	0.263	0.003	0.003	0.310
*Consultants working in surgical specialties*									
Emergency	0.00096	0.0022	0.657	0.001	0.003	0.816	0.000	0.004	0.964
Elective	0.000	0.000	0.950	0.001	0.000	0.009	0.001	0.001	0.048

Regression coefficients used for risk-adjustment are reported in Additional file 8 and Additional file 9.

## Discussion

Medical revalidation in the UK has reformed regulation of the profession, in response to earlier regulatory failures. Its implementation has been closely observed by regulators of other professions and by medical regulators around the world. Assessing its implementation, including any unintended consequences that result, is of considerable policy interest.

The introduction of medical revalidation in 2012 was associated with an increased risk of hospital consultants subsequently ceasing NHS clinical practice. This finding applies across all age groups. Consultants awaiting their first revalidation recommendation were at higher risk of leaving, and the hazard further increased after a deferral or non-engagement recommendation. A positive revalidation recommendation reduced the risk but was still associated with an increased risk of exit compared with pre-policy levels for older consultants. Other contemporaneous changes to the labour market and working environment (e.g. organisational change in the NHS, including those made as a result of the Health and Social Care Act [22] or the reduction of the limit on UK pension savings from 2012 onwards [23]) may also have led to an increased risk of exit independent of the introduction of medical revalidation. The observed increase in the hazard of exit while consultants await their first revalidation recommendation is likely to be confounded by these contemporaneous, external influences. But, under the assumption that the hazard of exit following a positive recommendation fully reflects these confounding influences, the difference between the hazards in the ‘preparing for revalidation’ and ‘positive recommendation’ states may be interpreted as a causal effect of the policy intervention. Difference-in-differences analysis does not support the hypothesis of an increase in mortality rates of consultants who left practice compared with those who stayed in practice after the introduction of medical revalidation in 2012.

This research has several important strengths compared with earlier reports of the effect of medical revalidation. Firstly, we use a data source that reflects actual NHS practice, rather than the GMC register, which does not differentiate between clinically active doctors and those that hold a licence but no longer practise. Secondly, we use robust quasi-experimental methods and detailed data. Linking HES with the GMC register permits us to assess the likelihood of ceasing clinical activity, the characteristics of doctors who do and of the patients they treat. Doctors were issued with different revalidation dates and although these were not necessarily random we exploited this variation to estimate robustly the effect of the policy, reducing the risk of confounding by other events. Finally, using information on patient health outcomes before and after the introduction of medical revalidation allowed us to assess the performance of consultants who ceased practice following revalidation compared with those who remain.

There are nevertheless a number of limitations to the analysis. First, we focus solely on hospital consultants (fully trained specialists), not doctors in training, those in primary care or other settings. Second, scheduled revalidation dates were not made available to us and are therefore, in our datasets, unobserved for doctors that left practice before their revalidation meeting, or for which revalidation is scheduled to occur after the end of our data window. We observe the date of revalidation only for those who completed the process and received a recommendation. Third, some consultants may stop taking charge of care episodes but still provide care as part of a wider team. This may induce measurement error, especially if consultants subsequently stopped working in an NHS trust before February 2017. Fourth, as our data are derived from Hospital Episodes Statistics, consultants are viewed as ceasing practice if they no longer take charge of care episodes in English NHS hospitals—they may continue to work in non-clinical roles or in other hospitals (e.g. in the private sector, or in other countries of the UK) or in primary care. Finally, 30-day mortality rates have been criticised as imperfect measures of healthcare quality, especially when case-mix adjustment is restricted to the limited information collected in administrative databases [24].

Our findings are consistent with concerns expressed in qualitative studies and surveys, and with early evidence from the GMC that doctors were relinquishing their licence to practice following the introduction of medical revalidation [12, 15, 16]. Ongoing qualitative research [17, 25] further informs the reasons for this apparent change in the likelihood of consultants ceasing NHS practice. These reports suggest that the administrative burden and inflexibility has had an effect, but there may also be more fundamental questions about whether mechanisms that monitor performance can undermine morale in healthcare professionals [3]. We are not aware of any other research exploring the performance of consultants leaving the profession in response to medical revalidation.

The overall size of the consultant workforce is increasing despite the higher risk of consultants leaving. This is because of expansions in medical school intake since 1997, which have resulted in increased fully trained consultants over recent years [26, 27]. The increased risk of ceasing NHS practice may be a one-off effect, and longer term research will be required to determine this.

## Conclusion

The introduction of medical revalidation in England increased the risk of hospital consultants ceasing clinical activity. There is no evidence that those ceasing NHS practice provided, on average, lower quality care, as measured by patient mortality within 30 days of admission.

## Additional files

Additional file 1: Association of consultants’ case-load in 2008 on risk of ceasing activity. Figure shows estimated hazard ratios (HRs) and 95% confidence intervals (CIs) for different case-load groups relative to the group with lowest case-load in 2008. (PDF 60 kb)

Additional file 2: Stratified analysis of time to exit by consultant age. Hazard ratios (HRs) and 95% confidence intervals (CIs). (PDF 557 kb)

Additional file 3: Stratified analysis of time to exit by consultant gender. Hazard ratios (HRs) and 95% confidence intervals (CIs). (PDF 530 kb)

Additional file 4: Stratified analysis of time to exit by country of primary medical qualification. Hazard ratios (HRs) and 95% confidence intervals (CIs). (PDF 529 kb)

Additional file 5: Sensitivity analyses of length of follow-up and type of parametric model. Hazard ratios (HRs) and 95% confidence intervals (CIs). (PDF 582 kb)

Additional file 6: Tabulation of adjusted and unadjusted 30-day mortality rate by leaver/stayer status and time period. Hazard ratios (HRs) and 95% confidence intervals (CIs). (PDF 516 kb)

Additional file 7: Difference in mortality rates between leavers and stayers by specialty and time period; outcomes are assigned to first consultant in admission spell. (PDF 504 kb)

Additional file 8: Association between risk factors and mortality - consultant works in medical specialty. Odds ratios (ORs) and 95% confidence intervals (CIs) for the risk of dying within 30 days of admissions for patients treated in medical specialties. Coefficients on HRG indicators are available from the authors on request. See Section “Statistical analysis” of the manuscript for details about variable definition and model specification. (PDF 405 kb)

Additional file 9: Association between risk factors and mortality - consultant works in surgical specialty. Odds ratios (ORs) and 95% confidence intervals (CIs) for the risk of dying within 30 days of admissions for patients treated in surgical specialties. Coefficients on HRG indicators are available from the authors on request. See Section “Statistical analysis” of the manuscript for details about variable definition and model specification. (PDF 405 kb)
