# Supplementary figures and images for: Does regulation increase the rate at which doctors leave practice? Analysis of routine hospital data in the English NHS following the introduction of medical revalidation
Source: BMC Med. 2019 Feb 11;17:33. doi: 10.1186/s12916-019-1270-4 (PMC6371486; doi:10.1186/s12916-019-1270-4)

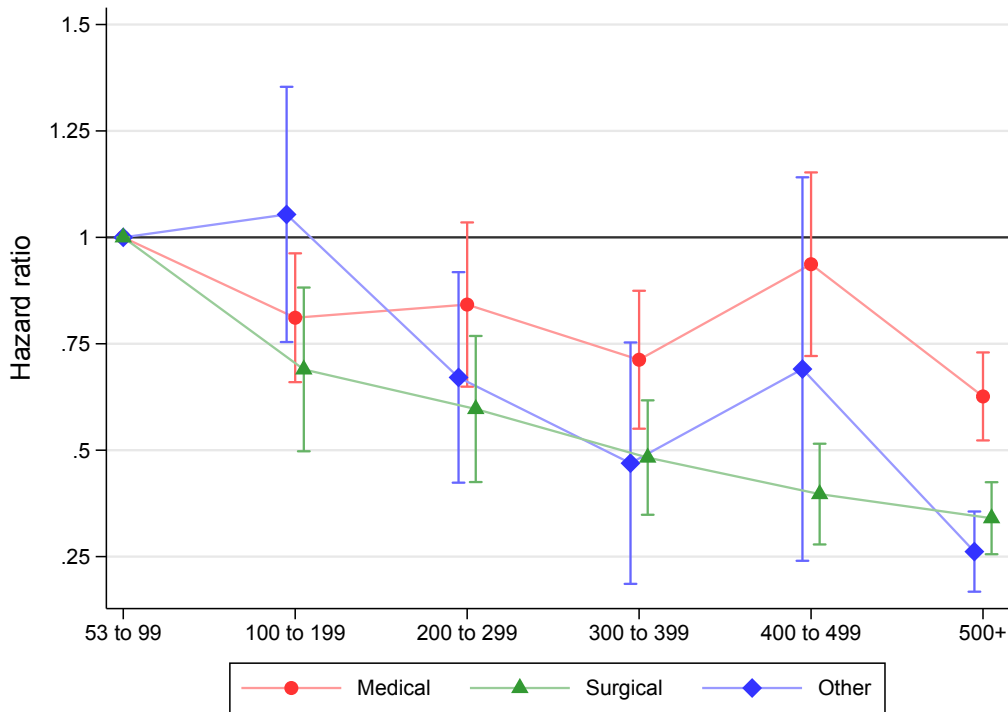

Supplement: Supplementary file 1 — Association of consultants’ case-load in 2008 on risk of ceasing activity. Figure shows estimated hazard ratios (HRs) and 95% confidence intervals (CIs) for different case-load groups relative to the group with lowest case-load in 2008. (PDF 60 kb) [file 12916_2019_1270_MOESM1_ESM.pdf]
