# Supplementary material for: Does regulation increase the rate at which doctors leave practice? Analysis of routine hospital data in the English NHS following the introduction of medical revalidation
Source: BMC Med. 2019 Feb 11;17:33. doi: 10.1186/s12916-019-1270-4 (PMC6371486; doi:10.1186/s12916-019-1270-4)
Supplement: Supplementary file 2 — Stratified analysis of time to exit by consultant age. Hazard ratios (HRs) and 95% confidence intervals (CIs). (PDF 557 kb) [file 12916_2019_1270_MOESM2_ESM.pdf]

Stratified analysis by consultant age

	Age <=40			Age 41-45			Age 46-50			Age 51-55			Age 56-60			Age 61-65			Age >65		
	HR	95% CI		HR	95% CI		HR	95% CI		HR	95% CI		HR	95% CI		HR	95% CI		HR	95% CI	
Specialty																					
Medical	(base category)			(base category)			(base category)			(base category)			(base category)			(base category)			(base category)		
Other	1.63	0.89	2.97	1.05	0.56	1.97	2.63	1.54	4.49	3.30	1.96	5.57	2.61	1.51	4.54	0.80	0.39	1.65	0.64	0.32	1.31
Surgical	1.52	0.65	3.57	0.81	0.35	1.87	3.03	1.24	7.40	2.28	1.08	4.81	2.48	1.12	5.48	2.07	1.08	3.98	1.00	0.52	1.91
Volume of activity in 2008																					
53-99	(base category)			(base category)			(base category)			(base category)			(base category)			(base category)			(base category)		
100-199	1.15	0.65	2.04	0.43	0.22	0.83	0.81	0.49	1.33	1.04	0.64	1.71	0.97	0.58	1.63	0.76	0.50	1.17	0.61	0.26	1.43
200-299	0.75	0.37	1.52	0.74	0.43	1.28	0.64	0.33	1.22	0.98	0.57	1.70	1.30	0.78	2.18	0.76	0.47	1.25	0.48	0.22	1.04
300-399	0.47	0.18	1.26	0.65	0.36	1.17	0.84	0.46	1.55	0.64	0.33	1.22	1.06	0.66	1.71	0.69	0.41	1.16	0.35	0.10	1.30
400-499	0.90	0.44	1.82	0.81	0.45	1.44	0.93	0.49	1.75	1.23	0.69	2.19	1.38	0.79	2.41	0.71	0.42	1.18	0.59	0.24	1.46
>=500	0.54	0.31	0.93	0.52	0.34	0.78	0.57	0.35	0.90	0.69	0.45	1.08	0.92	0.61	1.39	0.60	0.42	0.85	0.32	0.15	0.66
Volume x Specialty																					
Other x 100-199	0.86	0.37	1.99	2.75	0.94	8.05	0.81	0.38	1.74	0.81	0.43	1.53	0.95	0.40	2.29	2.82	1.08	7.34	6.73	2.03	22.28
Other x 200-299	0.45	0.10	2.03	1.92	0.53	6.97	0.68	0.21	2.24	0.36	0.13	1.03	0.54	0.24	1.19	2.31	0.81	6.55	0.00	0.00	0.00
Other x 300-399	1.22	0.14	10.88	0.00	0.00	0.00	0.26	0.03	2.10	1.24	0.38	3.99	0.47	0.15	1.49	0.86	0.26	2.86	0.00	0.00	0.00
Other x 400-499	0.58	0.07	4.67	0.00	0.00	0.00	0.00	0.00	0.00	0.52	0.09	3.22	0.57	0.18	1.74				17.54	4.79	64.28
Other x >=500	0.84	0.26	2.74	0.48	0.10	2.20	0.19	0.04	0.81	0.08	0.03	0.26	0.29	0.14	0.61	1.25	0.52	2.99	0.00	0.00	0.00
Surgical x 100-199	0.61	0.20	1.81	3.75	1.28	10.96	0.89	0.29	2.78	0.52	0.20	1.34	0.52	0.19	1.41	0.71	0.30	1.65	1.81	0.66	5.00
Surgical x 200-299	0.90	0.28	2.87	2.08	0.80	5.44	0.45	0.16	1.31	0.45	0.19	1.05	0.38	0.15	0.97	0.60	0.28	1.27	2.29	0.78	6.77
Surgical x 300-399	1.11	0.31	3.91	1.29	0.41	4.05	0.30	0.10	0.92	0.64	0.23	1.75	0.56	0.24	1.33	0.52	0.23	1.17	0.98	0.21	4.63
Surgical x 400-499	0.38	0.11	1.29	0.87	0.31	2.48	0.24	0.08	0.75	0.27	0.11	0.67	0.31	0.13	0.76	0.42	0.19	0.92	1.54	0.42	5.71
Surgical x >=500	0.58	0.22	1.52	1.20	0.50	2.89	0.27	0.11	0.69	0.37	0.17	0.82	0.39	0.18	0.84	0.51	0.27	0.96	2.27	0.88	5.87
Country of primary medical qualification																					
UK trained	(base category)			(base category)			(base category)			(base category)			(base category)			(base category)			(base category)		
Foreign trained	3.71	2.59	5.31	1.96	1.41	2.72	1.53	1.04	2.25	1.26	0.97	1.63	0.93	0.74	1.18	1.00	0.82	1.23	0.63	0.40	0.98
Consultant gender																					
Male	(base category)			(base category)			(base category)			(base category)			(base category)			(base category)			(base category)		
Female	0.91	0.71	1.17	0.95	0.73	1.23	1.03	0.76	1.39	1.31	1.07	1.60	1.27	1.07	1.52	1.20	0.94	1.53	2.12	1.10	4.08
Revalidation status																					
Pre-policy - not subject to revalidation	(base category)			(base category)			(base category)			(base category)			(base category)			(base category)			(base category)		
Post-policy - awaiting revalidation	1.94	1.42	2.64	2.73	2.12	3.52	2.85	2.21	3.66	2.52	1.97	3.23	1.94	1.62	2.34	1.88	1.55	2.29	2.45	1.68	3.56
Post-policy - deferred/non-engagement	0.87	0.13	5.99	6.55	3.67	11.69	5.53	2.65	11.52	3.26	1.70	6.27	2.92	1.79	4.75	1.47	0.76	2.81	3.81	0.99	14.69
Post-policy - revalidated	1.02	0.67	1.56	1.30	0.91	1.87	2.12	1.55	2.90	2.35	1.82	3.05	1.35	1.09	1.68	1.57	1.20	2.04	2.24	1.02	4.89
N	3336			5032			4250			3259			2172			1117			168		
