# Supplementary material for: Does regulation increase the rate at which doctors leave practice? Analysis of routine hospital data in the English NHS following the introduction of medical revalidation
Source: BMC Med. 2019 Feb 11;17:33. doi: 10.1186/s12916-019-1270-4 (PMC6371486; doi:10.1186/s12916-019-1270-4)
Supplement: Supplementary file 3 — Stratified analysis of time to exit by consultant gender. Hazard ratios (HRs) and 95% confidence intervals (CIs). (PDF 530 kb) [file 12916_2019_1270_MOESM3_ESM.pdf]

Stratified analysis by consultant gender

	Male			Female		
	HR	95% CI		HR	95% CI	
Specialty						
Medical	(base category)			(base category)		
Other	1.61	1.21	2.14	2.27	1.44	3.59
Surgical	1.65	1.20	2.27	2.05	0.98	4.31
Volume of activity in 2008						
53-99	(base category)			(base category)		
100-199	0.83	0.66	1.04	0.72	0.46	1.11
200-299	0.83	0.64	1.06	0.88	0.57	1.35
300-399	0.70	0.54	0.90	0.76	0.47	1.22
400-499	0.88	0.67	1.16	1.11	0.71	1.75
>=500	0.61	0.50	0.73	0.69	0.50	0.96
Volume x Specialty						
Other x 100-199	1.25	0.82	1.91	1.60	0.82	3.13
Other x 200-299	0.96	0.58	1.60	0.50	0.24	1.03
Other x 300-399	0.51	0.19	1.33	1.06	0.44	2.58
Other x 400-499	1.03	0.48	2.19	0.31	0.07	1.49
Other x >=500	0.41	0.26	0.65	0.41	0.22	0.78
Surgical x 100-199	0.86	0.58	1.26	0.87	0.33	2.24
Surgical x 200-299	0.74	0.51	1.07	0.66	0.28	1.58
Surgical x 300-399	0.71	0.48	1.04	0.62	0.23	1.68
Surgical x 400-499	0.47	0.32	0.69	0.22	0.08	0.63
Surgical x >=500	0.56	0.40	0.78	0.69	0.32	1.51
Country of primary medical qualification						
UK trained	(base category)			(base category)		
Foreign trained	1.22	1.09	1.37	1.75	1.39	2.19
Consultant age (in 2008)						
<=40	(base category)			(base category)		
41-45	1.21	1.01	1.45	1.00	0.70	1.43
46-50	1.37	1.11	1.68	1.04	0.69	1.56
51-55	1.97	1.57	2.48	1.44	0.91	2.28
56-60	3.01	2.33	3.90	2.05	1.16	3.63
61-65	3.90	2.91	5.22	2.06	1.00	4.20
>65	3.56	2.44	5.21	3.61	1.00	12.98
Revalidation status						
Pre-policy - not subject to revalidation	(base category)			(base category)		
Post-policy - awaiting revalidation	2.41	2.18	2.67	2.01	1.63	2.48
Post-policy - deferred/non-engagement	3.43	2.58	4.58	3.67	2.04	6.61
Post-policy - revalidated	1.90	1.68	2.14	1.60	1.23	2.09
N	15386			3948		
