# Supplementary material for: Does regulation increase the rate at which doctors leave practice? Analysis of routine hospital data in the English NHS following the introduction of medical revalidation
Source: BMC Med. 2019 Feb 11;17:33. doi: 10.1186/s12916-019-1270-4 (PMC6371486; doi:10.1186/s12916-019-1270-4)
Supplement: Supplementary file 4 — Stratified analysis of time to exit by country of primary medical qualification. Hazard ratios (HRs) and 95% confidence intervals (CIs). (PDF 529 kb) [file 12916_2019_1270_MOESM4_ESM.pdf]

Stratified analysis by country of primary medical qualification

	UK trained			Foreign trained		
	HR	95% CI		HR	95% CI	
Specialty						
Medical	(base category)			(base category)		
Other	2.27	1.70	3.02	1.42	1.00	2.03
Surgical	1.88	1.33	2.65	1.52	0.93	2.50
Volume of activity in 2008						
53-99	(base category)			(base category)		
100-199	0.94	0.75	1.17	0.60	0.42	0.86
200-299	0.91	0.70	1.19	0.68	0.47	0.97
300-399	0.75	0.56	1.00	0.67	0.46	0.98
400-499	1.00	0.77	1.29	0.83	0.56	1.23
>=500	0.65	0.54	0.80	0.58	0.44	0.78
Volume x Specialty						
Other x 100-199	1.24	0.83	1.86	1.33	0.75	2.35
Other x 200-299	0.67	0.42	1.08	0.95	0.46	1.95
Other x 300-399	0.65	0.32	1.30	0.73	0.26	2.01
Other x 400-499	0.64	0.29	1.43	0.51	0.06	4.35
Other x >=500	0.28	0.18	0.45	0.73	0.40	1.34
Surgical x 100-199	0.76	0.51	1.15	1.14	0.61	2.14
Surgical x 200-299	0.62	0.40	0.95	1.00	0.55	1.82
Surgical x 300-399	0.62	0.40	0.97	0.86	0.46	1.61
Surgical x 400-499	0.39	0.26	0.60	0.51	0.27	0.95
Surgical x >=500	0.55	0.39	0.78	0.52	0.31	0.90
Consultant age (in 2008)						
<=40	(base category)			(base category)		
41-45	1.07	0.80	1.44	0.93	0.71	1.21
46-50	0.94	0.64	1.37	0.92	0.69	1.22
51-55	1.03	0.65	1.65	1.23	0.92	1.66
56-60	1.32	0.78	2.24	1.95	1.42	2.70
61-65	1.50	0.84	2.69	3.57	2.47	5.16
>65	1.62	0.80	3.25	2.96	1.82	4.82
Consultant gender						
Male	(base category)			(base category)		
Female	0.77	0.55	1.10	1.13	0.82	1.57
Revalidation status						
Pre-policy - not subject to revalidation	(base category)			(base category)		
Post-policy - awaiting revalidation	2.08	1.85	2.34	2.50	2.18	2.87
Post-policy - deferred/non-engagement	2.72	1.96	3.77	4.72	3.19	6.97
Post-policy - revalidated	1.60	1.39	1.85	1.89	1.57	2.28
N	13487			5847		
