# Supplementary material for: Does regulation increase the rate at which doctors leave practice? Analysis of routine hospital data in the English NHS following the introduction of medical revalidation
Source: BMC Med. 2019 Feb 11;17:33. doi: 10.1186/s12916-019-1270-4 (PMC6371486; doi:10.1186/s12916-019-1270-4)
Supplement: Supplementary file 5 — Sensitivity analyses of length of follow-up and type of parametric model. Hazard ratios (HRs) and 95% confidence intervals (CIs). (PDF 582 kb) [file 12916_2019_1270_MOESM5_ESM.pdf]

Sensitivity analyses - length of follow-up and type of parametric model

	Follow-up until									Parametric model														
	30/11/2014			31/03/2015			31/03/2015 (HES only)			Exponential			Weibull			Gompertz			Log-Normal			Log-Logistic		
	HR	95% CI		HR	95% CI		HR	95% CI		HR	95% CI		HR	95% CI		HR	95% CI		HR	95% CI		HR	95% CI	
Specialty																								
Medical		(base category)			(base category)			(base category)			(base category)			(base category)			(base category)			(base category)			(base category)	
Other	1.96	1.46	2.64	1.82	1.40	2.36	3.19	2.51	4.05	1.63	1.30	2.05	1.63	1.30	2.05	1.64	1.30	2.08	0.53	0.38	0.76	0.75	0.67	0.86
Surgical	1.93	1.37	2.72	1.74	1.28	2.36	1.60	1.19	2.14	1.72	1.30	2.28	1.72	1.30	2.28	1.82	1.37	2.42	0.59	0.37	0.95	0.80	0.67	0.97
Volume of activity in 2008																								
53-99		(base category)			(base category)			(base category)			(base category)			(base category)			(base category)			(base category)			(base category)	
100-199	0.75	0.59	0.97	0.79	0.64	0.98	0.78	0.65	0.94	0.82	0.69	0.97	0.82	0.69	0.97	0.81	0.68	0.97	1.31	1.00	1.73	1.12	1.01	1.23
200-299	0.67	0.50	0.91	0.79	0.62	1.00	0.76	0.61	0.96	0.83	0.67	1.03	0.83	0.67	1.03	0.84	0.67	1.05	1.55	1.10	2.18	1.15	1.02	1.28
300-399	0.69	0.52	0.93	0.65	0.50	0.83	0.62	0.49	0.79	0.72	0.58	0.89	0.72	0.58	0.89	0.70	0.56	0.88	1.64	1.14	2.35	1.19	1.06	1.35
400-499	0.95	0.71	1.26	0.92	0.72	1.18	0.80	0.63	1.03	0.91	0.73	1.13	0.91	0.73	1.13	0.90	0.72	1.13	1.20	0.84	1.70	1.07	0.95	1.21
>=500	0.53	0.42	0.67	0.57	0.47	0.68	0.53	0.44	0.63	0.60	0.51	0.70	0.60	0.51	0.70	0.60	0.51	0.70	2.13	1.54	2.94	1.32	1.20	1.45
Volume x Specialty																								
Other x 100-199	1.27	0.86	1.87	1.24	0.85	1.81	1.14	0.82	1.58	1.31	0.95	1.82	1.31	0.95	1.82	1.39	1.00	1.95	0.87	0.54	1.42	0.94	0.80	1.12
Other x 200-299	0.73	0.41	1.28	0.82	0.53	1.27	0.72	0.49	1.05	0.81	0.54	1.22	0.81	0.54	1.21	0.84	0.56	1.25	1.04	0.53	2.08	1.11	0.89	1.40
Other x 300-399	0.50	0.21	1.22	0.69	0.32	1.48	0.49	0.25	0.95	0.58	0.31	1.09	0.58	0.31	1.09	0.59	0.31	1.13	1.80	0.73	4.45	1.24	0.88	1.74
Other x 400-499	0.86	0.38	1.93	0.67	0.28	1.60	0.65	0.30	1.38	0.77	0.40	1.48	0.77	0.40	1.48	0.76	0.40	1.47	2.02	0.76	5.36	1.42	1.02	1.97
Other x >=500	0.44	0.26	0.72	0.36	0.24	0.56	0.21	0.14	0.32	0.45	0.30	0.66	0.45	0.30	0.66	0.45	0.31	0.67	2.81	1.57	5.02	1.53	1.24	1.91
Surgical x 100-199	0.87	0.59	1.29	0.86	0.60	1.23	0.89	0.63	1.26	0.86	0.62	1.20	0.86	0.62	1.20	0.83	0.59	1.17	1.05	0.60	1.84	1.02	0.82	1.26
Surgical x 200-299	0.69	0.44	1.08	0.69	0.48	1.00	0.75	0.52	1.08	0.70	0.50	0.98	0.70	0.50	0.98	0.66	0.47	0.93	1.18	0.71	1.98	1.12	0.91	1.37
Surgical x 300-399	0.56	0.35	0.90	0.69	0.47	1.02	0.72	0.49	1.04	0.64	0.45	0.91	0.64	0.45	0.91	0.62	0.44	0.88	1.54	0.88	2.68	1.21	0.98	1.50
Surgical x 400-499	0.39	0.25	0.61	0.43	0.30	0.63	0.47	0.33	0.68	0.41	0.29	0.57	0.41	0.29	0.57	0.39	0.28	0.55	2.78	1.52	5.08	1.49	1.20	1.85
Surgical x >=500	0.47	0.33	0.68	0.54	0.39	0.74	0.56	0.41	0.75	0.53	0.39	0.71	0.53	0.39	0.71	0.50	0.38	0.68	1.96	1.19	3.23	1.33	1.09	1.61
Country of primary medical qualification																								
UK trained		(base category)			(base category)			(base category)			(base category)			(base category)			(base category)			(base category)			(base category)	
Foreign trained	1.25	1.10	1.42	1.23	1.10	1.38	1.25	1.12	1.39	0.96	0.88	1.04	0.96	0.86	1.06	1.29	1.16	1.43	0.64	0.58	0.71	0.68	0.65	0.72
Consultant age (in 2008)																								
<=40		(base category)			(base category)																			
41-45	1.12	0.86	1.47	1.15	0.93	1.42	1.18	0.98	1.42	1.05	0.88	1.25	1.05	0.88	1.26	0.90	0.75	1.07	1.02	0.87	1.19	1.13	1.06	1.20
46-50	1.20	0.92	1.58	1.23	0.99	1.54	1.18	0.97	1.43	1.19	1.00	1.42	1.19	0.98	1.45	0.86	0.71	1.04	1.01	0.85	1.19	1.25	1.17	1.33
51-55	1.51	1.13	2.01	1.61	1.26	2.04	1.47	1.18	1.82	2.42	2.06	2.83	2.43	1.99	2.95	1.45	1.18	1.78	0.50	0.35	0.72	1.08	1.00	1.16
56-60	2.69	1.92	3.76	2.60	1.99	3.41	2.29	1.77	2.96	6.36	5.43	7.45	6.40	5.17	7.91	3.15	2.49	3.99	0.08	0.03	0.21	0.73	0.65	0.82
61-65	3.80	2.59	5.56	3.41	2.48	4.69	2.84	2.10	3.84	12.34	10.42	14.60	12.42	9.70	15.89	5.05	3.80	6.72	0.00	0.00	0.04	0.35	0.23	0.52
>65	3.61	2.26	5.75	3.24	2.17	4.82	2.55	1.70	3.84	13.45	10.54	17.15	13.55	9.80	18.74	4.30	2.90	6.38	0.00	0.00	0.05	0.34	0.26	0.44
Consultant gender																								
Male		(base category)			(base category)			(base category)			(base category)			(base category)			(base category)			(base category)			(base category)	
Female	1.01	0.76	1.34	0.91	0.70	1.18	1.11	0.89	1.38	0.84	0.66	1.07	0.84	0.66	1.07	0.84	0.66	1.07	1.12	0.92	1.36	1.07	0.99	1.16
Revalidation status																								
Pre-policy - not subject to revalidation		(base category)			(base category)			(base category)			(base category)			(base category)			(base category)			(base category)			(base category)	
Post-policy - awaiting revalidation	1.61	1.43	1.81	1.98	1.80	2.18	2.08	1.90	2.27	2.67	2.44	2.91	2.67	2.43	2.92	2.38	2.17	2.62	0.36	0.26	0.49	0.72	0.69	0.76
Post-policy - deferred/non-engagement	0.57	0.08	4.13	2.53	1.72	3.72	2.95	2.08	4.18	4.14	3.23	5.29	4.14	3.23	5.30	3.63	2.82	4.67	0.15	0.07	0.31	0.58	0.49	0.67
Post-policy - revalidated	1.14	0.83	1.57	1.37	1.20	1.57	1.73	1.54	1.95	2.20	1.98	2.45	2.21	1.97	2.47	1.89	1.69	2.12	0.50	0.39	0.64	0.83	0.79	0.87
Baseline risk																								
Constant										0.0000277	0.0000226	0.0000341	0.99	0.79	1.25	1.00	1.00	1.00	1.04	0.86	1.27	0.29	0.27	0.31
N	19334			19334			19334			19334			19334			19334			19334			19334		
