# Supplementary material for: Does regulation increase the rate at which doctors leave practice? Analysis of routine hospital data in the English NHS following the introduction of medical revalidation
Source: BMC Med. 2019 Feb 11;17:33. doi: 10.1186/s12916-019-1270-4 (PMC6371486; doi:10.1186/s12916-019-1270-4)
Supplement: Supplementary file 6 — Tabulation of adjusted and unadjusted 30-day mortality rate by leaver/stayer status and time period. Hazard ratios (HRs) and 95% confidence intervals (CIs). (PDF 516 kb) [file 12916_2019_1270_MOESM6_ESM.pdf]

Tabulation of unadjusted 30-day mortality rates, by leaver/stayer status and time period

Note: Outcomes are assigned to last consultant in hospital stay

	Medical - emergency				Medical - elective				Surgical - emergency				Surgical - elective			
	Rate	SE	95% CI		Rate	SE	95% CI		Rate	SE	95% CI		Rate	SE	95% CI	
Pre-policy																
Stayer	0.1008	0.0002	0.1005	0.1012	0.0118	0.0001	0.0117	0.0119	0.0441	0.0002	0.0436	0.0445	0.0033	0.00003	0.0033	0.0034
Leaver	0.1022	0.0020	0.0983	0.1062	0.0116	0.0007	0.0103	0.0130	0.0383	0.0017	0.0349	0.0416	0.0036	0.0004	0.0028	0.0044
Difference	0.0014	0.0020	-0.0026	0.0054	-0.0001	0.0007	-0.0014	0.0012	-0.0058	0.0017	-0.0092	-0.0024	0.0003	0.0004	-0.0005	0.0011
Post-policy																
Stayer	0.0961	0.0002	0.0957	0.0965	0.0103	0.0001	0.0102	0.0104	0.0401	0.0002	0.0397	0.0406	0.0027	0.00003	0.0027	0.0028
Leaver	0.1094	0.0019	0.1057	0.1132	0.0124	0.0006	0.0112	0.0136	0.0421	0.0025	0.0372	0.0471	0.0034	0.0003	0.0028	0.0041
Difference	0.0133	0.0019	0.0096	0.0171	0.0021	0.0006	0.0009	0.0033	0.0020	0.0025	-0.0030	0.0070	0.0007	0.0003	0.0001	0.0013

Tabulation of case-mix adjusted 30-day mortality rates, by leaver/stayer status and time period

Note: Outcomes are assigned to last consultant in hospital stay

	Medical - emergency				Medical - elective				Surgical - emergency				Surgical - elective			
	Rate	SE	95% CI		Rate	SE	95% CI		Rate	SE	95% CI		Rate	SE	95% CI	
Pre-policy																
Stayer	0.1013	0.0005	0.1004	0.0009	0.0122	0.0003	0.0116	0.0006	0.0455	0.0004	0.0448	0.0007	0.0036	0.00004	0.0035	0.0001
Leaver	0.0999	0.0040	-0.0078	0.0078	0.0120	0.0018	-0.0035	0.0035	0.0464	0.0021	-0.0041	0.0041	0.0036	0.0004	-0.0008	0.0008
Difference	-0.0014	0.0040	-0.0092	0.0065	-0.0003	0.0018	-0.0092	0.0065	0.0010	0.0021	-0.0092	0.0065	0.00003	0.0004	-0.0092	0.0065
Post-policy																
Stayer	0.0967	0.0005	0.0958	0.0977	0.0108	0.0002	0.0104	0.0113	0.0419	0.0004	0.0412	0.0426	0.0030	0.00004	0.0029	0.0031
Leaver	0.1059	0.0082	-0.0161	0.0161	0.0140	0.0028	-0.0055	0.0055	0.0427	0.0033	-0.0065	0.0065	0.0043	0.0005	-0.0010	0.0010
Difference	0.0091	0.0082	-0.0070	0.0253	0.0031	0.0028	-0.0070	0.0253	0.0008	0.0033	-0.0070	0.0253	0.0013	0.0005	-0.0070	0.0253
