# Supplementary material for: Does regulation increase the rate at which doctors leave practice? Analysis of routine hospital data in the English NHS following the introduction of medical revalidation
Source: BMC Med. 2019 Feb 11;17:33. doi: 10.1186/s12916-019-1270-4 (PMC6371486; doi:10.1186/s12916-019-1270-4)
Supplement: Supplementary file 7 — Difference in mortality rates between leavers and stayers by specialty and time period; outcomes are assigned to first consultant in admission spell. (PDF 504 kb) [file 12916_2019_1270_MOESM7_ESM.pdf]

Difference in mortality rates between leavers and stayers by specialty and time period; outcomes are assigned to first consultant in admission spell

	Difference pre-policy			Difference post-policy			Difference-in-difference		
Index admission	Est	SE	P-value	Est	SE	P-value	Est	SE	P-value
**Unadjusted comparison**									
Consultants working in medical specialties									
Emergency	0.001	0.002	0.498	0.013	0.002	<0.001	0.012	0.003	<0.001
Elective	0.000	0.001	0.856	0.002	0.001	<0.001	0.002	0.001	0.015
Consultants working in surgical specialties									
Emergency	-0.006	0.002	<0.001	0.002	0.003	0.430	0.008	0.003	0.011
Elective	0.000	0.000	0.491	0.001	0.000	0.029	0.000	0.001	0.403
**Risk-adjusted comparison**									
Consultants working in medical specialties									
Emergency	-0.001	0.004	0.733	0.009	0.008	0.268	0.011	0.009	0.252
Elective	0.000	0.002	0.887	0.003	0.003	0.263	0.003	0.003	0.310
Consultants working in surgical specialties									
Emergency	0.001	0.002	0.657	0.001	0.003	0.816	0.000	0.004	0.964
Elective	0.000	0.000	0.950	0.001	0.000	0.009	0.001	0.001	0.048
