# Supplementary material for: Does regulation increase the rate at which doctors leave practice? Analysis of routine hospital data in the English NHS following the introduction of medical revalidation
Source: BMC Med. 2019 Feb 11;17:33. doi: 10.1186/s12916-019-1270-4 (PMC6371486; doi:10.1186/s12916-019-1270-4)
Supplement: Supplementary file 8 — Association between risk factors and mortality - consultant works in medical specialty. Odds ratios (ORs) and 95% confidence intervals (CIs) for the risk of dying within 30 days of admissions for patients treated in medical specialties. Coefficients on HRG indicators are available from the authors on request. See Section “Statistical analysis” of the manuscript for details about variable definition and model specification. (PDF 405 kb) [file 12916_2019_1270_MOESM8_ESM.pdf]

Regression results - consultant works in medical specialty

	Emergency a				Elective admissions			
	Year 2010		Year 2013		Year 2010		Year 2013	
Covariate	OR	95% CI	OR	95% CI	OR	95% CI	OR	95% CI
Monday				(Base category)				
Tuesday	0.960	(0.945 to 0.976)	0.978	(0.960 to 0.997)	0.970	(0.908 to 1.035)	0.965	(0.910 to 1.023)
Wednesday	0.949	(0.933 to 0.965)	0.977	(0.958 to 0.995)	0.952	(0.921 to 0.985)	0.953	(0.918 to 0.990)
Thursday	0.935	(0.920 to 0.951)	0.967	(0.950 to 0.986)	0.971	(0.909 to 1.037)	0.940	(0.886 to 0.998)
Friday	0.949	(0.934 to 0.965)	0.974	(0.956 to 0.993)	1.024	(0.989 to 1.059)	0.999	(0.962 to 1.038)
Saturday	1.055	(1.037 to 1.074)	1.062	(1.041 to 1.083)	0.935	(0.819 to 1.067)	0.968	(0.869 to 1.079)
Sunday	1.092	(1.073 to 1.111)	1.101	(1.079 to 1.123)	1.059	(0.918 to 1.221)	1.128	(0.990 to 1.285)
Age 60-64				(Base category)				
Age 65-69	1.182	(1.157 to 1.207)	1.175	(1.146 to 1.205)	1.079	(0.951 to 1.223)	1.100	(0.977 to 1.238)
Age 70-74	1.376	(1.348 to 1.405)	1.357	(1.324 to 1.391)	1.327	(1.145 to 1.538)	1.248	(1.112 to 1.399)
Aged 75-79	1.641	(1.607 to 1.676)	1.589	(1.550 to 1.630)	1.379	(1.190 to 1.597)	1.432	(1.272 to 1.612)
Aged 80-84	2.038	(1.993 to 2.084)	2.002	(1.951 to 2.055)	1.832	(1.638 to 2.047)	1.631	(1.429 to 1.861)
Aged 85-89	2.590	(2.529 to 2.653)	2.549	(2.481 to 2.619)	2.000	(1.639 to 2.440)	2.002	(1.715 to 2.337)
Female				(Base category)				
Male	1.158	(1.146 to 1.169)	1.151	(1.138 to 1.165)	1.197	(1.117 to 1.283)	1.279	(1.206 to 1.356)
Comorbid conditions: 0				(Base category)				
Comorbid conditions: 1	1.259	(1.204 to 1.316)	1.251	(1.209 to 1.295)	1.111	(1.035 to 1.194)	1.162	(1.028 to 1.315)
Comorbid conditions: 2-3	1.697	(1.622 to 1.775)	1.745	(1.686 to 1.805)	1.455	(1.359 to 1.557)	1.369	(1.227 to 1.529)
Comorbid conditions: 4-6	2.319	(2.213 to 2.430)	2.396	(2.313 to 2.483)	1.740	(1.609 to 1.882)	1.758	(1.552 to 1.991)
Comorbid conditions: >6	1.993	(1.904 to 2.086)	2.121	(2.047 to 2.197)	1.453	(1.302 to 1.620)	1.378	(1.218 to 1.559)
Emergency admission in last year: no				(Base category)				
Emergency admission in last year: yes	0.876	(0.866 to 0.886)	0.882	(0.870 to 0.894)	0.852	(0.804 to 0.904)	0.860	(0.820 to 0.901)
Consultant: stayer				(Base category)				
Consultant: leaver	0.983	(0.891 to 1.085)	1.122	(0.922 to 1.366)	0.978	(0.718 to 1.332)	1.300	(0.864 to 1.956)
N	2,526,540		1,929,189		3,197,076		2,954,541	
