# Supplementary material for: Does regulation increase the rate at which doctors leave practice? Analysis of routine hospital data in the English NHS following the introduction of medical revalidation
Source: BMC Med. 2019 Feb 11;17:33. doi: 10.1186/s12916-019-1270-4 (PMC6371486; doi:10.1186/s12916-019-1270-4)
Supplement: Supplementary file 9 — Association between risk factors and mortality - consultant works in surgical specialty. Odds ratios (ORs) and 95% confidence intervals (CIs) for the risk of dying within 30 days of admissions for patients treated in surgical specialties. Coefficients on HRG indicators are available from the authors on request. See Section “Statistical analysis” of the manuscript for details about variable definition and model specification. (PDF 405 kb) [file 12916_2019_1270_MOESM9_ESM.pdf]

Regression results - consultant works in surgical specialty

	Emergency a				Elective admissions			
	Year 2010		Year 2013		Year 2010		Year 2013	
Covariate	OR	95% CI	OR	95% CI	OR	95% CI	OR	95% CI
Monday				(Base category)				
Tuesday	0.979	(0.943 to 1.017)	0.960	(0.917 to 1.005)	1.023	(0.959 to 1.091)	1.008	(0.930 to 1.093)
Wednesday	0.977	(0.941 to 1.015)	0.976	(0.932 to 1.022)	0.959	(0.897 to 1.026)	0.949	(0.875 to 1.030)
Thursday	0.984	(0.947 to 1.022)	0.962	(0.920 to 1.007)	0.985	(0.921 to 1.053)	1.053	(0.971 to 1.142)
Friday	0.952	(0.916 to 0.990)	0.941	(0.898 to 0.986)	0.963	(0.892 to 1.039)	0.964	(0.882 to 1.053)
Saturday	1.083	(1.041 to 1.127)	1.072	(1.022 to 1.125)	0.869	(0.742 to 1.018)	0.919	(0.785 to 1.075)
Sunday	1.121	(1.078 to 1.166)	1.040	(0.991 to 1.092)	1.144	(1.030 to 1.272)	1.116	(0.977 to 1.276)
Age 60-64				(Base category)				
Age 65-69	1.239	(1.178 to 1.303)	1.286	(1.207 to 1.369)	1.345	(1.225 to 1.476)	1.350	(1.206 to 1.510)
Age 70-74	1.515	(1.445 to 1.589)	1.645	(1.549 to 1.747)	1.801	(1.650 to 1.966)	1.691	(1.517 to 1.885)
Aged 75-79	2.033	(1.942 to 2.128)	2.085	(1.968 to 2.210)	2.350	(2.156 to 2.561)	2.207	(1.980 to 2.459)
Aged 80-84	2.823	(2.699 to 2.952)	2.893	(2.734 to 3.061)	3.388	(3.107 to 3.695)	3.123	(2.803 to 3.480)
Aged 85-89	4.065	(3.883 to 4.256)	4.156	(3.923 to 4.404)	4.923	(4.484 to 5.406)	4.516	(4.013 to 5.082)
Female				(Base category)				
Male	1.198	(1.169 to 1.227)	1.173	(1.140 to 1.207)	1.423	(1.356 to 1.494)	1.428	(1.347 to 1.513)
Comorbid conditions: 0				(Base category)				
Comorbid conditions: 1	1.404	(1.336 to 1.475)	1.484	(1.393 to 1.581)	1.224	(1.135 to 1.319)	1.283	(1.164 to 1.415)
Comorbid conditions: 2-3	2.276	(2.171 to 2.386)	2.340	(2.202 to 2.486)	1.865	(1.741 to 1.997)	1.827	(1.679 to 1.988)
Comorbid conditions: 4-6	3.443	(3.272 to 3.623)	3.722	(3.489 to 3.970)	2.861	(2.650 to 3.090)	2.745	(2.500 to 3.014)
Comorbid conditions: >6	2.498	(2.372 to 2.632)	2.774	(2.599 to 2.961)	1.903	(1.764 to 2.052)	1.842	(1.676 to 2.025)
Emergency admission in last year: no				(Base category)				
Emergency admission in last year: yes	0.800	(0.779 to 0.822)	0.821	(0.795 to 0.848)	0.787	(0.744 to 0.831)	0.835	(0.784 to 0.890)
Consultant: stayer				(Base category)				
Consultant: leaver	1.024	(0.922 to 1.137)	1.022	(0.855 to 1.220)	1.007	(0.805 to 1.261)	1.446	(1.143 to 1.829)
N	897,096		661,728		2,764,521		2,199,115	
